# Giant sigmoid diverticulum with coexisting metastatic rectal carcinoma: a case report

**DOI:** 10.1186/1752-1947-4-324

**Published:** 2010-10-18

**Authors:** Walid Sasi, Issam Hamad, Aidan Quinn, Abdul Rahman Nasr

**Affiliations:** 1 Department of Surgery, Louth County Hospital, Dundalk, Co Louth, Ireland; 2 Department of Radiology, Louth County Hospital, Dundalk, Co. Louth, Ireland

## Abstract

**Introduction:**

Giant diverticulum of the colon is a rare but clinically significant condition, usually regarded as a complication of an already existing colonic diverticular disease. This is the first report of a giant diverticulum of the colon with a co-existing rectal carcinoma.

**Case presentation:**

We report a case of a 66-year-old Caucasian woman who presented with lower abdominal pain, chronic constipation and abdominal swelling. Preoperative abdominal computed tomography revealed a giant diverticulum of the colon with a coexisting rectal carcinoma and pulmonary metastasis revealed on a further thoracic computed tomography. An *en bloc *anterior resection of the rectum along with sigmoid colectomy, partial hysterectomy and right salpingoophorectomy was subsequently performed due to extensive adhesions.

**Conclusion:**

This report shows that the presence of a co-existing distal colorectal cancer can potentially lead to progressive development of a colonic diverticulum to become a giant diverticulum by increasing colonic intra-luminal pressure and through the ball-valve mechanism. This may be of interest to practising surgeons and surgical trainees.

## Introduction

Giant diverticulum of the colon (GDC) is a rare but clinically significant condition, usually regarded as a complication of an already existing colonic diverticular disease. The etiology is not clearly understood but it occurs most frequently as a single giant diverticulum in the sigmoid colon and can present with a variety of symptoms and signs. Interestingly, there has been no previous report in the literature of this condition with a coexisting rectal carcinoma. In this article, we present the first published report of a patient with giant sigmoid diverticulum and a concomitant metastatic rectal carcinoma.

## Case presentation

A 66-year-old Caucasian (Irish) woman presented to our surgical outpatient clinic with lower abdominal pain, chronic constipation and abdominal swelling. She is an ex-smoker with a background history of diverticular disease and long-standing psoriasis.

**Figure 1:**
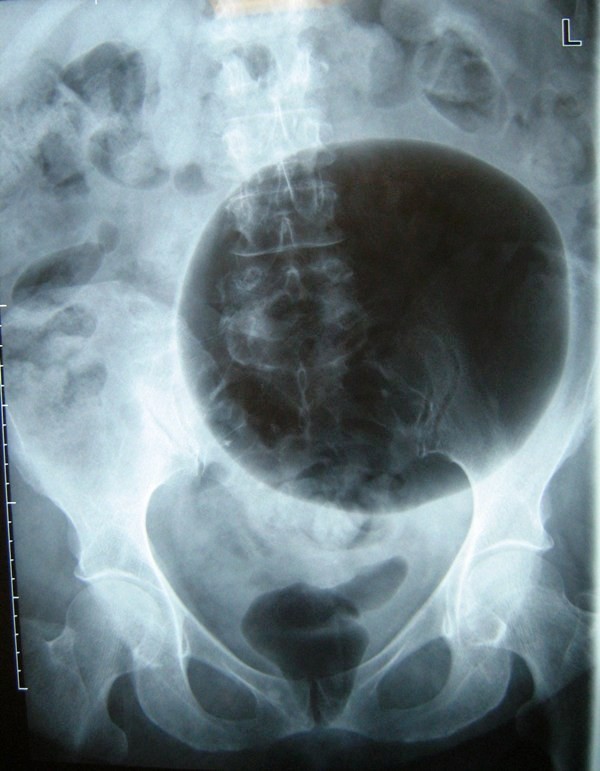
**An abdominal X-ray showing air filled giant diverticulum**.

**Figure 2:**
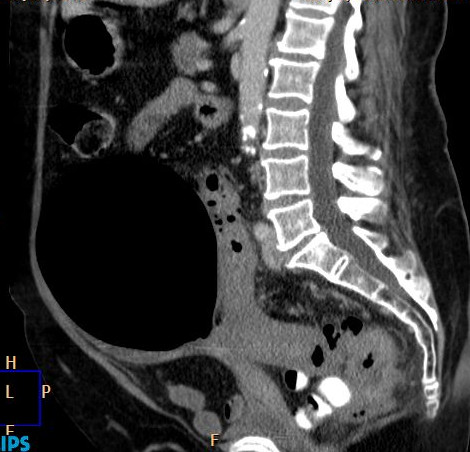
**A giant colonic diverticulum on computed tomography**.

Clinical examination on presentation revealed a large, slightly tender, left-sided abdominal mass which was tympanic on percussion. Baseline blood tests were all normal. A plain film of the abdomen showed a large air-filled cyst displacing bowel loops (Figure 1). A chest X-ray showed an ill-defined nodular opacity projected over the posterior segment of the right lung lower lobe. Subsequently, an abdominal computed tomography (CT) scan showed a communicating GDC of 14 cm size with multiple small diverticulae in the sigmoid colon along with an irregular thickening of the upper rectal wall highly suspicious of malignancy (Figure 2). A further CT scan of her thorax revealed multiple small nodules in both lung fields which were consistent with metastatic deposits. Fine needle aspirate from one of these nodules showed evidence of metastatic mucinous adenocarcinoma, probably from the rectal site.

**Figure 3:**
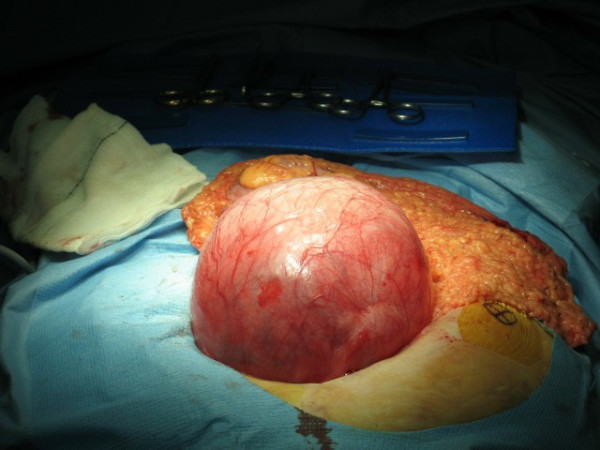
**A giant sigmoid diverticulum on laparotomy**.

**Figure 4:**
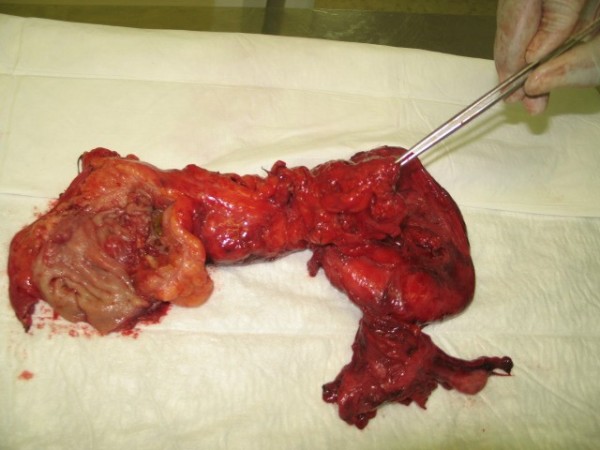
**A resected colorectal segment showing a deflated giant sigmoid diverticulum and rectal carcinoma (opened)**.

Her case was discussed in our departmental meeting and the decision was made to perform anterior resection of the rectum along with sigmoid colectomy. At surgery, a single GDC was found in the sigmoid colon with dense adhesions to the uterine fundus, the right ovary, the right fallopian tube and the posterior abdominal wall (Figures 3 and 4). Significant diverticular disease was also found along with a solid upper rectal tumor.

Anterior resection of the rectum together with *en bloc *sigmoid colectomy, partial hysterectomy and right salpingo-opherectomy was performed (Figure 4) and a colostomy was fashioned. It was not possible to perform colorectal anastomosis due to the considerable inflammation and adhesions. Macroscopic examination of the specimen revealed a thickened sigmoid wall with many diverticula and a large cyst of 14 cm diameter. The cyst wall was 1 mm to 10 mm thick, with irregular inner and smooth outer surfaces. There was a communicating stalk attaching to the bowel wall. In the rectum, there was an ulcerating tumor with a crater of 4 × 1.5 cm invading into the perirectal fat and reaching the peritoneum. Six lymph nodes and two conglomerates of lymph nodes were present along with a uterine corpus of four cm and right adnexa. Histological examination revealed a partly mucinous adenocarcinoma of the rectum extending to the peritoneal surface, with lymphovascular invasion and lymph node metastasis (Duke's C, T4N2M1) and diverticulosis of the sigmoid colon with a single GDC. The uterus was not involved in the malignant process but was very adherent to the bowel.

The postoperative recovery was uneventful and the colostomy had started to function on the second postoperative day. The patient was subsequently discharged home in a good condition and is now under joint surgical and oncological care.

## Discussion

Giant diverticulum of the colon (GDC) is a rare condition, with only less than 180 cases discussed in the literature since it was first reported by Bonvin and Bonte in 1946 [1]. It has been reported in different parts of the colon but in 81% of cases it occurred in the sigmoid colon and in nearly 90% of cases; there has been only one report of a single giant colonic diverticulum [2]. Most GDCs are diagnosed in elderly patients with mean age of presentation between 60 and 79 years. Its size has been most frequently reported in the range of 4-9 cm and rarely above 25 cm [2].

The etiology of GDC is not clearly understood, although in over 90% of reported cases there has been associated colonic diverticulosis [2]. Histologically, there are three described types of GDC, namely the true congenital diverticulum, where the wall has all the colonic structural layers, the pseudo-diverticulum, where the wall is mainly composed of mucosa, and the inflammatory GDC, where the wall is only a reactive scar tissue. The last type of GDC occurs as a result of a previous colonic perforation, mostly due to diverticular disease. A ball-valve mechanism has been suggested by Nano *et al. *as a cause of a gradual increase in the size of a colonic diverticulum until it transforms into GDC [3].

Higher pressures in the colon cause higher pressures inside the GDC by allowing air to pass through a one-way communicating stalk. Differences in the colonic pressure can also lead to differences in the GDC pressure leading to intermittently prominent abdominal mass or phantom tumor [3].

Clinical features of GDC can be variable. While some patients remain asymptomatic for long periods of time, many others present with chronic symptoms of abdominal pain, constipation, abdominal distension or weight loss. Still others may have acute presentation with abdominal pain, diarrhea, fever, nausea and vomiting or rectal bleeding. The most significant finding on clinical examination is an abdominal mass which is reported in nearly 60% of patients [2].

The investigations of choice for diagnosing GDC include a plain abdominal X-ray and an abdominal CT scan: both can accurately demonstrate the classical 'balloon sign' of GDC. Barium enema is useful in showing a communication with the bowel in most cases.

The two main complications are perforation and abscess formation. Among the less frequent complications is intestinal obstruction [4,5], intestinal volvulus [6], lower gastrointestinal bleeding [7] and lymphoma or adenocarcinoma arising within GDC [2,8].

To our knowledge, there have been no previous reports in the literature describing a coexisting rectal or distal colonic carcinoma along with GDC. The presence of a distal colorectal tumour can lead - in theory - to increased air pressure in GDC by the ball-valve mechanism described above if the tumor is large enough to cause colonic luminal narrowing and not necessarily by colonic obstruction. Our patient complained of chronic constipation but had no clinical features of intestinal obstruction during the course of her illness. However, both the mechanism of the development of GDC and its relationship with rectal cancer are not the key problems. The screening and early detection of colorectal cancer in patients with colonic diverticular disease should be emphasised, because the symptoms and signs of both conditions are similar.

Two major surgical approaches are recommended for the treatment of GDCs: diverticulectomy or resection of the involved colonic segment [9]. Each can be combined with a protecting colostomy. However, the management of this case involved additional rectal resection due to a coexisting rectal malignancy and also involved a partial hysterectomy and salpingo-opherectomy due to the presence of dense adhesions.

## Conclusion

Giant diverticulum of the colon is a rare condition which usually occurs in patients suffering from a pre-existing diverticular disease. The best way to explain GDC progressive development is that of the ball-valve air mechanism, especially with increased colonic intra-luminal pressure. The presence of a coexisting distal colorectal cancer can potentially lead to the progressive development of a colonic diverticulum which may become a GDC. This case report may be of particular interest to practising surgeons and surgical trainees.

## Abbreviations

GDC: giant diverticulum of the colon; CT: computed tomography.

## Competing interests

The authors declare that they have no competing interests.

## Consent

Written informed consent was obtained from the patient for publication of this case report and accompanying images. A copy of the written consent is available for review by the Editor-in-Chief of this journal.

## Authors' contributions

WS conceived the study and wrote the manuscript and is the corresponding author. IH provided information about the patient's clinical course, took the photos of the case and shared in the editing of the radiology slides. AQ carried out the radiological investigations and shared in the editing of the radiology slides. ARN supervised the preliminary manuscript and edited the histopathological report. All authors read and approved the final manuscript.
